# AI policies for authors, reviewers, and editors at the *JCI* and *JCI Insight*

**DOI:** 10.1172/JCI214485

**Published:** 2026-11-16

**Authors:** Sarah Jackson, Corinne L. Williams, Oliver Eickelberg, Elizabeth M. McNally

Artificial intelligence (AI) tools are rapidly evolving and reshaping how scientists work, from writing to research design to data analysis and presentation. Likewise, every step in the life of a manuscript, including evaluation and the publication process, has the potential to be touched by AI. Here, we discuss the JCI Family of Journals’ policies and practices related to appropriate AI usage by authors, reviewers, and editors.

## Authors

The *JCI* and *JCI Insight* policies have recently been updated to require an explicit AI disclosure statement in all published manuscripts. We understand that many, if not most, of our authors have integrated AI tools into various aspects of their work, including, but not limited to, experimental design, data analysis, literature searches, writing, text translation, and preparation of figures and graphical abstracts. From the journals’ perspective, there should be no “penalty” for using AI; we simply want to understand how these tools were utilized and provide transparency for our readers. The disclosure must be clear about what tasks were performed and which AI tools were used. Importantly, we stress that authors are responsible for verifying the accuracy of AI-generated content. As amazing as AI tools can be, they do make errors, and their output often requires context and user judgement to verify or disregard. Authors are ultimately responsible for ensuring the accuracy of the submitted content.

## Reviewers

The *JCI* and *JCI Insight* continue to prohibit the use of AI tools by reviewers. First and foremost, the peer review process rests on the assumption that the reviewer will treat the manuscript as confidential and privileged information. Uploading a manuscript to an AI platform shares the manuscript, and therefore its content can be used in training the AI model, particularly when using a public cloud model. Two recent high-profile cases highlight some uncertainty regarding the boundaries of using AI for academic research. The basis for the discovery of virally encoded array-associated reverse transcriptases by Anthropic using Claude (1) was called into question by a computational biologist who studied these enzymes and had been using Claude for many years as part of his work (2). Similarly, when OpenAI announced it had solved one of the world’s most difficult math problems, the Navier-Stokes problem, a mathematician working on the same problem alleged that input from his group’s work may have been exploited by OpenAI (3). Although in both cases the AI companies deny that these researchers’ work was the basis of the discoveries, the controversies highlight unease in the research community about the confidentiality of information provided to AI tools and concerns about whether data shared with AI might alter future output from the model (2, 3)

What about the use of local or restricted/private AI environments? In principle, private AI is deployed in a secure environment that contains the content within a private cloud, whereas a local model runs directly on consumer-grade hardware. Yet it is still possible, even within such an environment, that the data and content of the manuscript could influence the output of the reviewer’s later use of the AI tool for their own work. This would violate our prohibition of using the content of unpublished manuscripts disclosed as part of the peer review process for the reviewer’s own research. Moreover, we as a Journal have no way of verifying what type of AI model a reviewer uses and what the associated risks are in terms of potentially breaching confidentiality.

Perhaps the most important reason that Editors do not want AI-generated reviews is that we ask reviewers to provide us their opinion. To our reviewers: We value your opinion as experts in the field! We recognize that reviewers are not necessarily versed in all aspects of a study; however, we do not need you to turn to AI tools to comment on content outside your domain of expertise. We are looking for your perspective on the merits of a manuscript based on your firsthand experience. No AI tool can replace your experience and expertise. If the Editors wanted and/or thought that AI tools were appropriate for reviewing a paper, we could do this on our own. In fact, at this time, we intentionally do not incorporate AI reviews in our evaluation process, and we do not want our reviewers to do so either. We recognize that conversations about AI in reviewing manuscripts will continue to shift over time, but let us be clear in our current stance: Reviewers for the *JCI* and *JCI Insight* may not use AI in evaluating manuscripts in the peer review process.

## Editors and journal staff

Just as authors and reviewers have responsibilities regarding AI usage, so do members of our Editorial Boards and journal staff. Like peer reviewers, *JCI* and *JCI Insight* Editors may not use AI tools in assessing a manuscript, even in a local or private environment, for all the reasons discussed above. As disclosed on our website, the journals use an AI-powered software from Proofig AI to evaluate images in manuscripts for duplication and manipulation. We do not analyze all submissions through this software, and instead this tool is applied to the limited group of previously revised manuscripts or manuscripts that we are considering inviting for revision. We have been assured that the data are private and not used to train the AI tools; however, we recognize that the same trust issues that we discuss above are relevant. We are aware that the arsenal of AI tools for publishers continues to expand, and we do not rule out responsibly incorporating other tools in the future. We anticipate testing or incorporating new tools if they add value beyond peer review and our current integrity checks. Our commitment to our authors is to be transparent about AI tool usage, and if other tools are adopted in the future, we will make this clear on our policy pages.

Following acceptance of manuscripts, most authors choose to rapidly publish their work in the In Press Preview format. However, for those who opt to wait for the copyediting and production processes to be complete prior to publication, the issues of confidentiality by the journal still apply. Currently, our copyediting staff may use AI tools to assist with verifying some required items in the Methods and Author contributions sections for manuscripts that have already been published as In Press Previews. Our author services team and production editors do not currently use AI tools to assist in their work at the journals. As with usage of AI by the Editors, should this change in the future, we will be transparent on our website in disclosing AI tools used by members of our staff.

## Concluding remarks

Our requirement for AI disclosure statements by authors is a first step in understanding how these tools are used by the research community. As the use of AI tools evolves and new tools are developed, we will continue to monitor responsible usage by those involved in evaluating papers at each step. AI is shifting how work is done across industries, not just science, and we are not alone in grappling with the promise and perils of these powerful tools. We endeavor to use AI tools effectively and responsibly, and to create policies that make sense for all members of our community.

## Manuscript information

### AI disclosure.

SJ used Google searches, which provide AI overviews, October 2, 2026.

### Acknowledgments.

We thank John Hawley and William Jackson for helpful discussions.

## Version 1 11/16/2026

Electronic publication
